# Associations of body roundness index and sugar-sweetened beverage consumption with psychological symptoms in adolescents: a multicenter cross-sectional survey based on Chinese adolescents aged 12–18 years old

**DOI:** 10.3389/fnut.2025.1505491

**Published:** 2025-03-10

**Authors:** Long Chen, Wei Zheng, Caiyun Wei, Jiayu Ling, Qingtao Kong

**Affiliations:** ^1^Key Laboratory of Adolescent Health Assessment and Exercise Intervention of Ministry of Education, East China Normal University, Shanghai, China; ^2^School of Physical Education and Health, East China Normal University, Shanghai, China; ^3^School of Physical Education and Sport Science, Fujian Normal University, Fuzhou, China; ^4^Department of Physical Education, Shanghai Ocean University, Shanghai, China

**Keywords:** body roundness index, sugar-sweetened beverage consumption, psychological symptoms, adolescents, cross-sectional assessment

## Abstract

**Background:**

Adolescent psychosocial symptoms continue to rise, negatively affecting academic performance and future achievement, and have become an important public health issue of common concern worldwide. However, few studies have been conducted on the association between body roundness index (BRI) and sugar-sweetened beverage (SSB) consumption and psychological symptoms in Chinese adolescents. The present study may provide implications for the intervention and prevention of psychological symptoms in Chinese adolescents.

**Methods:**

In this study, 47,520 adolescents aged 12–18 years from six geographic regions of China were assessed cross-sectionally for BRI, SSB consumption, and psychological symptoms in 2023. Independent samples t-tests, chi-square tests, logistic regression analyses, and ordered logistic regression analyses were used to compare and analyze the associations that existed between BRI and SSB consumption and psychological symptoms among Chinese adolescents.

**Results:**

The BRI of Chinese adolescents aged 12–18 years was (2.03 ± 0.94). The proportions of adolescents with SSB consumption ≤1 times/week, 2–3 times/week, and ≥ 4 times/week were 33.2, 52.0, and 14.7%, respectively, and the differences were statistically significant in comparison (*X*^2^ value of 597.860, *p* < 0.001). The prevalence of psychological symptoms among Chinese adolescents was 19.2%. The prevalence rates of emotional problems, behavioral problems, and social adjustment difficulties were 26.1, 25.2, and 16.1%, respectively. After adjusting for relevant covariates, ordered logistic regression analysis showed that with BRI quartiles Q1 and SSB consumption ≤1 times/week as the reference group, the adolescents in the group with BRI quartiles of Q4 and SSB consumption ≥4 times/week (OR = 2.01, 95% CI: 1.77–2.30) had the highest risk of developing psychological symptoms (*p* < 0.001).

**Conclusion:**

There is an association between BRI and SSB consumption with psychological symptoms in Chinese adolescents. Chinese adolescents with higher BRI and SSB consumption were at higher risk of developing psychological symptoms. In the future, the increase in BRI and SSB consumption should be effectively controlled to prevent or reduce the occurrence of psychological symptoms.

## 1 Introduction

In recent years, the prevalence of psychological symptoms among adolescents has continued to rise, with serious negative impacts on academic, social, and future achievements, and has become a common mental health problem in countries around the world (1). Studies show that the global prevalence of depressive symptoms among adolescents aged 10–19 years is 34% and that the prevalence of depressive symptoms among adolescents has risen from 24% in 2001 to 37% in 2020, and continues to show an increasing trend (2). It has also been observed that the prevalence of major depressive disorder among adolescents in the Americas increased from 8.7% in 2005 to 11.3% in 2014, and continues to show an increasing trend, posing a serious disease burden on American society (3). There are also studies confirming that the prevalence of psychological symptoms among Finnish adolescents is 31% and continues to increase, negatively affecting adolescent mental health development and academic performance, as well as posing a potential threat to adult mental health (4). Similarly, the problem of adolescent psychosomatic symptoms is spreading to developing countries. A survey of adolescents aged 14–17 years in Uganda, an African country, showed that 16.35% of adolescents reported major depressive symptoms, and almost one out of three girls interviewed (29.68%) reported moderate depressive symptoms, posing a serious threat to adolescent health development (5). China, also a developing country, is no exception. A survey of Chinese adolescents between the ages of 13–18 showed that 21.4% of Chinese adolescents had psychological symptoms, and called for targeted interventions against the influencing factors to reduce the incidence of psychological symptoms among adolescents (6). It was also found that the prevalence of major depressive symptoms among Chinese adolescents was 1.3%, which was equivalent to the world average, and called for the necessary measures to be taken to intervene and prevent it (7). The prevalence of adolescent psychological symptoms is increasing, posing a threat to adolescent health and future achievement. For this reason, it is important to investigate and study the various factors affecting adolescent psychosocial symptoms to prevent and intervene in a better way.

Adolescent psychological symptoms are influenced by a relatively large number of factors, but they are mainly closely associated with lifestyle and eating behavioral habits. With dramatic lifestyle changes, adolescents’ physical activity levels continue to decline, and static behavioral time and screen time continue to increase, posing a serious threat to adolescent mental health (8–10). Investigations have shown that obese adolescents have a higher risk of developing psychological symptoms compared to normal-weight individuals (11–13). In addition, there are some differences in the results for different indicators for evaluating adolescent obesity. Studies have shown that BRI, as an indicator different from conventional BMI or waist circumference, has high accuracy in identifying adolescent psychological symptoms and continues to gain attention from researchers (14). Studies have shown that the BRI better combines the effects of height and waist circumference to more accurately assess adolescents’ body composition and psychological symptoms compared to BMI and waist circumference metrics (15). However, a review of the literature shows that fewer studies have been conducted on the association between BRI and psychological symptoms in Chinese adolescents. Some of these studies focused on older adults, and the sample selection was limited, which could not fully confirm the association between BRI and psychological symptoms (16).

According to surveys, SSB consumption among adolescents is continuing to rise, with serious negative impacts on adolescent health (17). Studies have shown a strong association between increased SSB consumption and the development of various chronic diseases such as obesity, type 2 diabetes, cardiovascular disease, hypertension, and others (18–20). At the same time, increased SSB consumption can lead to a higher incidence of dental caries in adolescents (21). Some studies have also found an association between increased SSB consumption and the development of psychological symptoms in adolescents (10). A post-survey of Norwegian adolescents showed that increased SSB consumption in adolescents (OR = 1.49) had a significant influence on elevated psychological symptoms (22). A survey of Chinese adolescents also showed that adolescents with higher weekly SSB consumption (≥ 6 times/week) (OR = 3.06) had a significantly higher risk of psychological symptoms compared to those with lower SSB consumption (≤ 1 time/week) (23). This shows that it is necessary to intervene in the prevention of adolescent psychological symptoms by starting with the influencing factors, such as lowering SSB consumption and lowering body weight, to better prevent the occurrence of psychological symptoms.

Adolescence is the peak period of youthful development, a period of extreme psychological instability (24). Synthesizing previous relevant studies, it can be found that in the past, the effects of body composition, such as BMI and waist circumference, on psychological symptoms were mainly analyzed from a single perspective. BRI, as a new type of index for evaluating body composition, has been less studied about adolescents’ psychological symptoms (25). As a developing country, the prevalence of psychological symptoms among adolescents continues to increase, and there is a need to draw sufficient attention to this issue (26). In addition, to the best of our knowledge, no current studies have been found on the association between BRI and SSB consumption and psychological symptoms in Chinese adolescents. For this reason, the present study assessed BRI, SSB consumption, and psychological symptoms in 47,520 adolescents aged 12–18 years from six geographic regions in China. The associations between BRI and SSB consumption and psychological symptoms were analyzed. The aim is to provide reference and reference for the intervention and prevention of psychological symptoms in Chinese adolescents.

## 2 Methods

### 2.1 Participants

**Figure 1:**
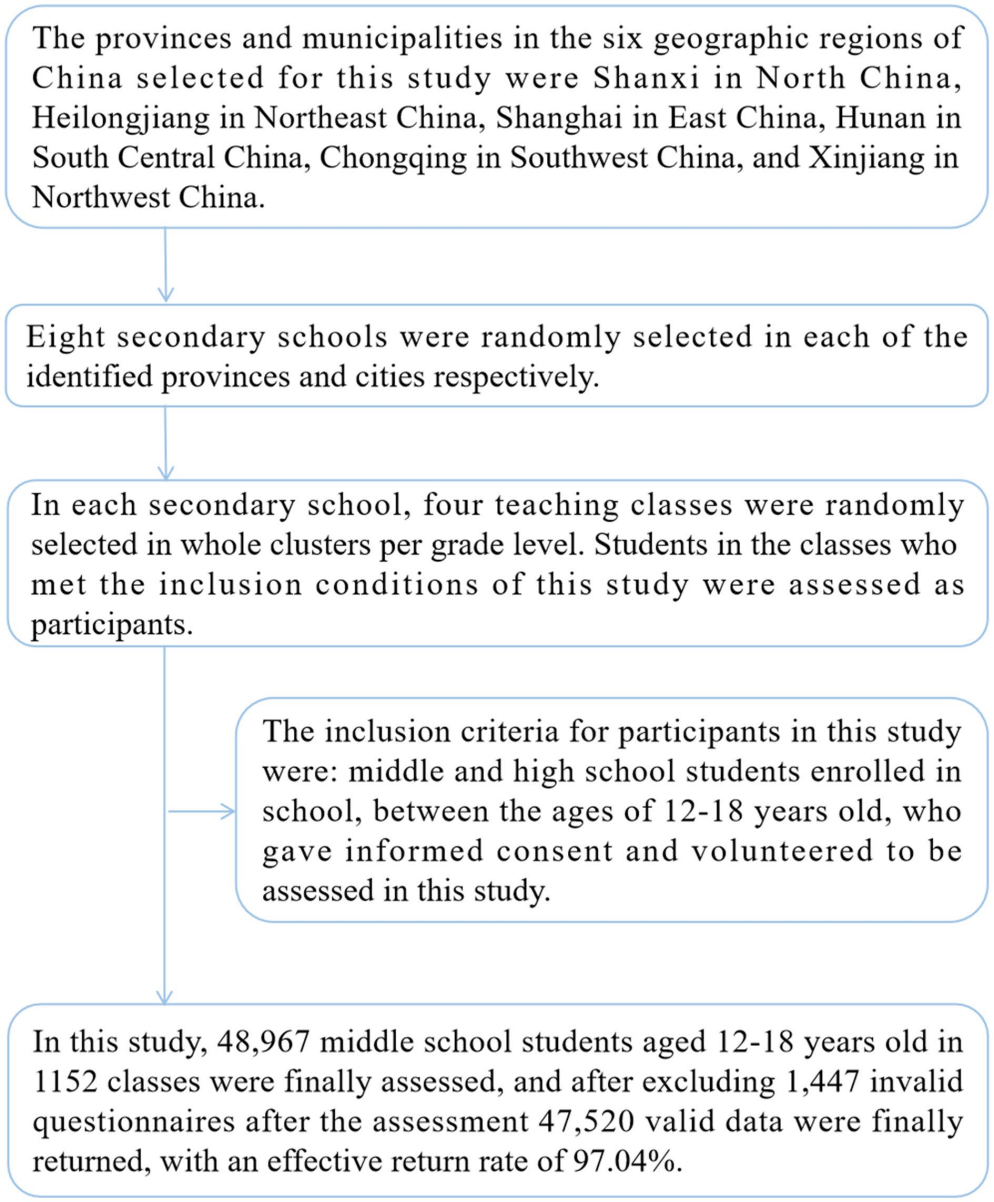
Sampling flow of Chinese adolescent participants.

In this study, 47,520 adolescents aged 12–18 years were assessed cross-sectionally for BRI, SSB consumption, and psychological symptoms from March to July 2023 in one province and city selected from each of the six geographic regions of China. Participants in this study were extracted in four stages. In the first stage, the provinces and municipalities in the six geographic regions of China selected for this study were Shanxi in North China, Heilongjiang in Northeast China, Shanghai in East China, Hunan in South Central China, Chongqing in Southwest China, and Xinjiang in Northwest China. In the second stage, eight secondary schools were randomly selected in each of the identified provinces and cities, respectively. In the third stage, in each secondary school, four teaching classes were randomly selected in whole clusters per grade level. In the fourth stage, students in the classes who met the inclusion conditions of this study were assessed as participants. The inclusion criteria for participants in this study were: middle and high school students enrolled in school, between the ages of 12–18 years old, who gave informed consent and volunteered to be assessed in this study. In this study, 48,967 middle school students aged 12–18 years old in 1152 classes were finally assessed, and after excluding 1,447 invalid questionnaires after the assessment, 47,520 valid data were finally returned, with an effective return rate of 97.04%. The participant extraction process of this study is shown in Figure 1.

This study was conducted by the Declaration of Helsinki. This study was approved by the Ethics Committee of Shanghai Ocean University (R8976436).

### 2.2 Psychological symptoms

In this study, psychological symptoms were assessed using the Brief Instrument on Psychological Health of Youths (BIOPHY) questionnaire (27). The BIOPHY consists of 15 entries categorized into 3 dimensions, namely, emotional problems, behavioral problems, and social adjustment difficulties. The scores for the three dimensions were summed to obtain a score for the psychological symptoms dimension. Each of the 15 entries was scored on a scale of 1–6 and recorded as a 1–6 score. The scores were “lasted 3 months and more,” “lasted 2 months,” “lasted 1 month,” “lasted 2 weeks,” “lasted 1 week,” and “lasted less than 1 week or none.” The higher the participant’s score the shorter the surface duration of the symptom, i.e., the shorter the duration of the psychological symptoms. Psychological symptoms were defined as present when participants’ symptoms lasted ≥1 month. Each of the 3-dimensional scores used P90 as a cutoff point for adolescent psychological symptoms problems, with <P90 defined as psychological symptoms and ≥ P90 defined as the absence of psychological symptoms. The BIOPHY has been used in several studies, and it can better evaluate the psychological symptoms of Chinese adolescents, with good reliability and validity. The Cronbach’s alpha coefficient for the total questionnaire of BIOPHY was 0.928, and the split-half coefficient was 0.909, indicating that the questionnaire had high internal consistency (28, 29).

### 2.3 Body roundness index (BRI)

The specific value of the BRI was based on the assessment of the participants’ waist circumference and height, which was then calculated by a formula. The specific BRI calculation formula is: BRI = 364.2–365.5*(1-[WC (m)/2π]^2^/[0.5*height (m)]^2^)½ (30). Participants’ waist circumference and height were assessed according to the testing instruments and methods required by the China National Survey on Students’ Constitution and Health (CNSSCH) (31). The results of both the waist circumference and height tests were accurate to 0.1 centimeters. Participants were asked to wear as light clothing as possible during the waist circumference assessment to minimize the error in the assessment. For height assessment, participants were asked to remove their shoes to ensure the accuracy of the test results (31).

### 2.4 Sugar-sweetened beverage (SSB)

Participants’ SSB consumption in this study was assessed using the frequency of weekly consumption as a criterion, categorized as ≤1 time/week, 2–3 times/week, and ≥ 4 times/week. SSB consumption was assessed using the beverage intake questionnaire (BEVQ-15) scale was used to assess SSB consumption (32). The BEVQ-15 scale consists of 15 entries, which mainly assesses participants’ SSB consumption in the past 30 days, including the frequency of SSB consumption, the number of milliliters, and the type of SSB. The BEVQ-15 scale has been widely used among Chinese adolescents, and it has good reliability and validity in assessing Chinese adolescents’ SSB consumption (28, 33). The types of SSBs in the BEVQ-15 scale include carbonated beverages, functional beverages, coffee, nut-based beverages, beverages with added sugar, unsweetened beverages, beer, wine, sweetened milk, all types of milk tea, and all types of beverages with added sugar that are not covered. Participants were asked to complete the questionnaire honestly based on their actual SSB consumption in the past 30 days. The frequency of SSB consumption was categorized as “<1 time per week” to “3 or more times per day” in the BEVQ-15 scale. The number of milliliters of SSB consumption was divided into five categories, ranging from “less than 6 ounces” to “20 ounces or more.” The SSB consumption was finally calculated based on the participants’ actual consumption by using the following formula (34). In this study, to facilitate the assessment of adolescent SSB consumption, the frequency of SSB consumption in the past 7 days was assessed using “8 ounces (1 cup)” as the assessment criterion, and participants were categorized as ≤1 times/week, 2–3 times/week, ≥4 times/week, and ≥ 3 times/week.

### 2.5 Covariates

Several past studies have shown that factors such as parental education, commuting to and from work, hours of sleep, physical activity, physical obesity, and muscle strength all have an impact on adolescent mental health (35–38). Based on previous studies, some covariates were included in the analysis in conjunction with this study. The covariates in this study include the father’s education、mother’s education, mode of commuting to and from school, sleep duration, MVPA, BMI, grip strength. Father’s education and mother’s education are divided into elementary school and below, middle school, high school, college and above. The mode of commuting to and from school is divided into active mode and negative mode. Active mode includes walking and bicycling; Negative mode includes taking the bus, taking the subway, private car transportation, and riding a battery-operated vehicle (31). Sleep duration is calculated based on the time of going to sleep and the time of waking up, and is classified as <7 h/day, 7–9 h/day, >9 h/day according to the relevant classification criteria (39). The MVPA is based on the assessment questionnaire in the CNSSCH (31). Calculations were primarily based on the duration and frequency of participants’ MVPA participation over the past 7 days, including Monday through Friday weekdays and Saturday and Sunday hours. In this study, MVPA was categorized as <30 min/day, 30–60 min/day, and > 60 min/day (31). The assessment of height, weight, waist circumference, and grip strength in this study was performed according to the methods and instruments required in the CNSSCH (31). BMI was calculated as weight (kg)/height (m)^2^.

### 2.6 Statistical analysis

Continuous variables in this study were expressed as mean and standard deviation (M ± SD). Categorical variables were expressed as percentages. Comparison of continuous type variables between boys and girls in this study was done using a t-test and categorical variables were compared using chi-square test. Different BRI types (Q1, Q2, Q3, Q4) and SSB consumption (≤1 times/week, 2–3 times/week, ≥4 times/week) psychological symptoms and dimensions (emotional problems, behavioral problems, and social adjustment difficulties) were compared using the chi-square test. In this study, Logistic regression analysis and ordered Logistic regression analysis were used to analyze the association between BRI and SSB consumption with psychological symptoms in adolescents. In Logistic regression analysis, the presence of psychological symptoms was used as the dependent variable, and BRI type and SSB consumption were used as the independent variables. Logistic regression analyses controlled for some of the covariates to account for covariance with BRI and SSB consumption. Model 1 is coarse. Model 2 adjusted age, father’s education, and mother’s education, based on Model 1. Model 3 adjusts the mode of commuting to and from school, sleep duration, and MVPA based on Model 2. In ordered Logistic regression analysis, the analytical model was adjusted age, father’s education, mother’s education, mode of commuting to and from school, sleep duration, and MVPA. Data analysis in this study was processed by SPSS25.0 software. *p* < 0.05 was used as the bilateral test level.

## 3 Results

This study assessed BRI and SSB consumption and psychological symptoms in 47,520 secondary school students aged 12–18 years in six geographic regions of China. The mean age of the participants was (15.12 ± 1.88) years.

Table 1 shows the basic situation of Chinese adolescents aged 12–18 years. The BRI of the participants was (2.03 ± 0.94). Boys’ (2.14 ± 1.02) BRI was higher than girls’ (1.93 ± 0.85), and the difference was statistically significant (*t* = 23.922, *p* < 0.001). The adolescents with SSB consumption ≤1 times/week, 2–3 times/week, and ≥ 4 times/week in this study were 33.2, 52.0, and 14.7%, respectively, and the difference was statistically significant compared to each other (*χ*^2^ value of 597.860, *p* < 0.001). The results of this study showed that the prevalence of psychological symptoms among Chinese adolescents was 19.2%. Among them, boys (19.8%) were higher than girls (18.5%), and the difference was statistically significant (*χ*^2^ value was 11.991, *p* < 0.01). The prevalence rates of emotional problems, behavioral problems, and social adjustment difficulties among Chinese adolescents were 26.1, 25.2, and 16.1%, respectively.

**Table 1:** Basic situation of adolescents aged 12–18 in China.

Items	Boys	Girls	Total	*χ*^2^ /*t*-value	*p*-value
Number	23,478	24,042	47,520		
Age (years)	15.05 ± 1.86	15.18 ± 1.89	15.12 ± 1.88	−7.955	<0.001
Father’s education [*N* (%)]					
Elementary school and below	2,682 (11.4)	2,821 (11.7)	5,503 (11.6)	3.808	0.283
Middle school	8,402 (35.8)	8,459 (35.2)	16,861 (35.5)		
High school	7,727 (32.9)	8,049 (33.5)	15,776 (33.2)		
College and above	4,667 (19.9)	4,713 (19.6)	9,380 (19.7)		
Mother’s education [*N* (%)]					
Elementary school and below	4,212 (17.9)	4,219 (17.5)	8,431 (17.7)	6.090	0.107
Middle School	7,975 (34.0)	8,335 (34.7)	16,310 (34.3)		
High School	7,144 (30.4)	7,398 (30.8)	14,542 (30.6)		
College and above	4,147 (17.7)	4,090 (17.0)	8,237 (17.3)		
Mode of commuting to and from school [*N* (%)]					
Active mode	11,383 (48.5)	10,509 (43.7)	21,892 (46.1)	108.901	<0.001
Negative mode	12,095 (51.5)	13,533 (56.3)	25,628 (53.9)		
Sleep duration [*N* (%)]					
<7 h/day	3,676 (15.7)	4,086 (17.0)	7,762 (16.3)	120.163	<0.001
7–9 h/day	16,223 (69.1)	17,103 (71.1)	33,326 (70.1)		
>9 h/day	3,579 (15.2)	2,853 (11.9)	6,432 (13.5)		
MVPA [*N* (%)]					
<30 min/day	8,943 (38.1)	13,101 (54.5)	22,044 (46.4)	1653.313	<0.001
30–60 min/day	10,082 (42.9)	8,767 (36.5)	18,849 (39.7)		
>60 min/day	4,453 (19.0)	2,174 (9.0)	6,627 (13.9)		
Height (M ± SD)	169.92 ± 9.05	161.17 ± 6.35	165.49 ± 8.95	122.310	<0.001
Weight (M ± SD)	59.67 ± 13.34	51.58 ± 9.11	55.58 ± 12.09	77.309	<0.001
BMI (M ± SD)	20.53 ± 3.68	19.82 ± 3.12	20.17 ± 3.43	22.574	<0.001
Waist circumference (M ± SD)	72.25 ± 11.20	66.57 ± 8.87	69.38 ± 10.48	61.446	<0.001
Grip strength (M ± SD)	35.83 ± 10.20	25.30 ± 6.31	30.50 ± 9.96	135.758	<0.001
BRI (M ± SD)	2.14 ± 1.02	1.93 ± 0.85	2.03 ± 0.94	23.933	<0.001
BRI Quartiles [*N* (%)]					
Q1	5,373 (22.9)	6,486 (27.0)	11,859 (25.0)	409.142	<0.001
Q2	5,505 (23.4)	6,387 (26.6)	11,892 (25.0)		
Q3	5,820 (24.8)	6,078 (25.3)	11,898 (25.0)		
Q4	6,780 (28.9)	5,091 (21.2)	11,871 (25.0)		
SSB Consumption [*N* (%)]					
≤1 times/week	6,734 (28.7)	9,049 (37.6)	15,783 (33.2)	597.860	<0.001
2–3 times/week	12,569 (53.5)	12,163 (50.6)	24,732 (52.0)		
≥4 times/week	4,175 (17.8)	2,830 (11.8)	7,005 (14.7)		
Emotional problems [*N* (%)]	6,162 (26.2)	6,253 (26.0)	12,415 (26.1)	0.346	0.556
Behavioral problems [*N* (%)]	6,222 (26.5)	5,737 (23.9)	11,959 (25.2)	43.926	<0.001
Social adjustment difficulties [*N* (%)]	4,022 (17.1)	3,646 (15.2)	7,668 (16.1)	33.920	<0.001
Psychological symptoms [*N* (%)]	4,648 (19.8)	4,459 (18.5)	9,107 (19.2)	11.991	0.001

*N, numbers; M, Mean; SD, standard deviation; BRI, body roundness index; SSB, sugar-sweetened beverage; MVPA, moderate-to-vigorous physical activity.*

Table 2 shows the comparison of psychological symptoms among Chinese adolescents with different BRI and SSB consumption. Overall, when comparing the prevalence of emotional problems, behavioral problems, social adjustment difficulties, and psychological symptoms among Chinese adolescents with different BRI quartiles, all differences were statistically significant (*χ*^2^-value 52,416, 65,294, 38,008, respectively). Chinese adolescents with SSB consumption ≤1 times/week, 2–3 times/week, and ≥ 4 times/week had similar prevalence rates of emotional problems, behavioral problems, and social adjustment difficulties, psychological symptoms, the differences were all statistically significant (*χ*^2^-value 165.368, 168.343, 175.329, 183.676, *p*-value <0.001). Overall, it can be seen that BRI quartiles for adolescents in group Q4 had a higher prevalence of psychological symptoms and all three dimensions than adolescents in group Q1. The prevalence of psychological symptoms and the three dimensions was higher in the SSB consumption ≥4 times/week group than in the ≤1 times/week group. The same trend was found in both male and female Chinese adolescents.

**Table 2:** Comparison of psychological symptoms among adolescents with different BRI and SSB consumption in China.

Gender/Category	Group	*N*	Emotional problems			Behavioral problems			Social adjustment difficulties			Psychological symptoms		
			*N* (%)	*χ*^2^-value	*p-*value	*N* (%)	*χ*^2^-value	*p*-value	*N* (%)	*χ*^2^-value	*p*-value	*N* (%)	*χ*^2^-value	*p*-value
Boys														
BRI quartiles	Q1	5,373	1,461 (27.2)	15.153	0.002	1,444 (26.9)	17.842	<0.001	979 (18.2)	11.357	0.010	927 (17.3)	42.845	<0.001
	Q2	5,505	1,374 (25.0)			1,428 (25.9)			917 (16.7)			1,081 (19.6)		
	Q3	5,820	1,468 (25.2)			1,447 (24.9)			933 (16.0)			1,148 (19.7)		
	Q4	6,780	1859 (27.4)			1903 (28.1)			1,193 (17.6)			1,492 (22.0)		
SSB consumption	≤1 times/week	6,734	1761 (26.2)	81.752	<0.001	1766 (26.2)	65.195	<0.001	1,169 (17.4)	82.022	<0.001	1,331 (19.8)	80.330	<0.001
	2–3 times/week	12,569	3,081 (24.5)			3,146 (25.0)			1951 (15.5)			2,290 (18.2)		
	≥4 times/week	4,175	1,320 (31.6)			1,310 (31.4)			902 (21.6)			1,027 (24.6)		
Girls														
BRI quartiles	Q1	6,486	1732 (26.7)	43.904	<0.001	1,611 (24.8)	53.614	<0.001	1,019 (15.7)	34.543	<0.001	1,119 (17.3)	58.721	<0.001
	Q2	6,387	1,497 (23.4)			1,354 (21.2)			859 (13.4)			1,097 (17.2)		
	Q3	6,078	1,560 (25.7)			1,409 (23.2)			890 (14.6)			1,116 (18.4)		
	Q4	5,091	1,464 (28.8)			1,363 (26.8)			878 (17.2)			1,127 (22.1)		
SSB consumption	≤1 times/week	9,049	2,284 (25.2)	87.743	<0.001	2075 (22.9)	98.827	<0.001	1,349 (14.9)	87.887	<0.001	1,637 (18.1)	103.186	<0.001
	2–3 times/week	12,163	3,028 (24.9)			2,775 (22.8)			1703 (14.0)			2,102 (17.3)		
	≥4 times/week	2,830	941 (33.3)			887 (31.3)			594 (21.0)			720 (25.4)		
Total														
BRI quartiles	Q1	11,859	3,193 (26.9)	52.416	<0.001	3,055 (25.8)	65.294	<0.001	1998 (16.8)	38.008	<0.001	2046 (17.3)	98.003	<0.001
	Q2	11,892	2,871 (24.1)			2,782 (23.4)			1776 (14.9)			2,178 (18.3)		
	Q3	11,898	3,028 (25.4)			2,856 (24.0)			1823 (15.3)			2,264 (19.0)		
	Q4	11,871	3,323 (28.0)			3,266 (27.5)			2071 (17.4)			2,619 (22.1)		
SSB consumption	≤1 times/week	15,783	4,045 (25.6)	165.368	<0.001	3,841 (24.3)	168.343	<0.001	2,518 (16.0)	175.329	<0.001	2,968 (18.8)	183.676	<0.001
	2–3 times/week	24,732	6,109 (24.7)			5,921 (23.9)			3,654 (14.8)			4,392 (17.8)		
	≥4 times/week	7,005	2,261 (32.3)			2,197 (31.4)			1,496 (21.4)			1747 (24.9)		

*N, numbers; BRI, body roundness index; SSB, sugar-sweetened beverage.*

Table 3 shows the logistic regression analysis of adolescents’ psychological symptoms with different BRI and SSB consumption. Binary logistic regression analyses were conducted with the presence of psychological symptoms in Chinese adolescents as the dependent variable and BRI and SSB consumption as the independent variables. Model 1 is coarse. Model 2 adjusted age, father’s education, and mother’s education, based on Model 1. Model 3 adjusts the mode of commuting to and from school, sleep duration, and MVPA based on Model 2. The analysis showed that overall, after adjusting for relevant covariates, the risk of psychological symptoms was significantly higher in adolescents in Q3 (OR = 1.14, 95% CI: 1.06–1.22) and Q4 (OR = 1.36, 95% CI: 1.27–1.45) groups, using the Q1 group of BRI quartiles as the reference group (*p* < 0.001). Using the SSB consumption ≤1 times/week group as the reference group, adolescents in the ≥4 times/week group (OR = 1.32, 95% CI: 1.22 ~ 1.42) also had a significantly higher risk of developing psychological symptoms (*p* < 0.001). Notably, compared with the SSB consumption ≤1 times/week group, adolescents in the Chinese adolescents with SSB consumption 2–3 times/week group (OR = 0.91, 95%CI: 0.86 ~ 0.96) had a lower risk of developing psychological symptoms (*p* < 0.001).

**Table 3:** Logistic regression analysis of psychological symptoms in Chinese adolescents with different BRI and SSB consumption.

Sex/Variable	Group	Psychological symptoms					
		Model 1		Model 2		Model 3	
		OR (95% CI)	*p-*value	OR (95% CI)	*p-*value	OR (95% CI)	*p-*value
Boys							
BRI quartiles	Q1	1.00		1.00		1.00	
	Q2	1.17 (1.06 ~ 1.29)	0.001	1.18 (1.07 ~ 1.30)	0.001	1.19 (1.07 ~ 1.31)	0.001
	Q3	1.18 (1.07 ~ 1.30)	0.001	1.19 (1.08 ~ 1.31)	<0.001	1.21 (1.10 ~ 1.33)	<0.001
	Q4	1.35 (1.24 ~ 1.48)	<0.001	1.39 (1.26 ~ 1.52)	<0.001	1.39 (1.27 ~ 1.53)	<0.001
SSB consumption	≤1 times/week	1.00		1.00		1.00	
	2–3 times/week	0.90 (0.84 ~ 0.98)	0.009	0.91 (0.84 ~ 0.98)	0.014	0.90 (0.83 ~ 0.97)	0.007
	≥4 times/week	1.32 (1.21 ~ 1.45)	<0.001	1.38 (1.26 ~ 1.51)	<0.001	1.23 (1.11 ~ 1.36)	<0.001
Girls							
BRI quartiles	Q1	1.00		1.00		1.00	
	Q2	1.00 (0.91 ~ 1.09)	0.908	1.00 (0.91 ~ 1.10)	0.990	1.01 (0.92 ~ 1.11)	0.851
	Q3	1.08 (0.98 ~ 1.18)	0.104	1.08 (0.98 ~ 1.18)	0.115	1.08 (0.99 ~ 1.19)	0.092
	Q4	1.36 (1.24 ~ 1.50)	<0.001	1.36 (1.24 ~ 1.49)	<0.001	1.35 (1.22 ~ 1.48)	<0.001
SSB consumption	≤1 times/week	1.00		1.00		1.00	
	2–3 times/week	0.95 (0.88 ~ 1.02)	0.126	0.94 (0.88 ~ 1.01)	0.117	0.90 (0.83 ~ 0.97)	0.004
	≥4 times/week	1.55 (1.40 ~ 1.71)	<0.001	1.6 (1.45 ~ 1.77)	<0.001	1.39 (1.25 ~ 1.55)	<0.001
Total							
BRI quartiles	Q1	1.00		1.00		1.00	
	Q2	1.08 (1.01 ~ 1.15)	0.032	1.08 (1.01 ~ 1.16)	0.022	1.08 (1.01 ~ 1.16)	0.019
	Q3	1.13 (1.06 ~ 1.20)	<0.001	1.13 (1.06 ~ 1.21)	<0.001	1.14 (1.06 ~ 1.22)	<0.001
	Q4	1.36 (1.27 ~ 1.45)	<0.001	1.37 (1.28 ~ 1.46)	<0.001	1.36 (1.27 ~ 1.45)	<0.001
SSB consumption	≤1 times/week	1.00		1.00		1.00	
	2–3 times/week	0.93 (0.89 ~ 0.98)	0.008	0.94 (0.89 ~ 0.98)	0.010	0.91 (0.86 ~ 0.96)	<0.001
	≥4 times/week	1.44 (1.34 ~ 1.53)	<0.001	1.49 (1.39 ~ 1.60)	<0.001	1.32 (1.22 ~ 1.42)	<0.001

*N, numbers; BRI, body roundness index; SSB, sugar-sweetened beverage. Model 1 is coarse. Model 2 adjusted age, father’s education, and mother’s education, based on Model 1. Model 3 adjusts the mode of commuting to and from school, sleep duration, and MVPA based on Model 2.*

**Figure 2:**
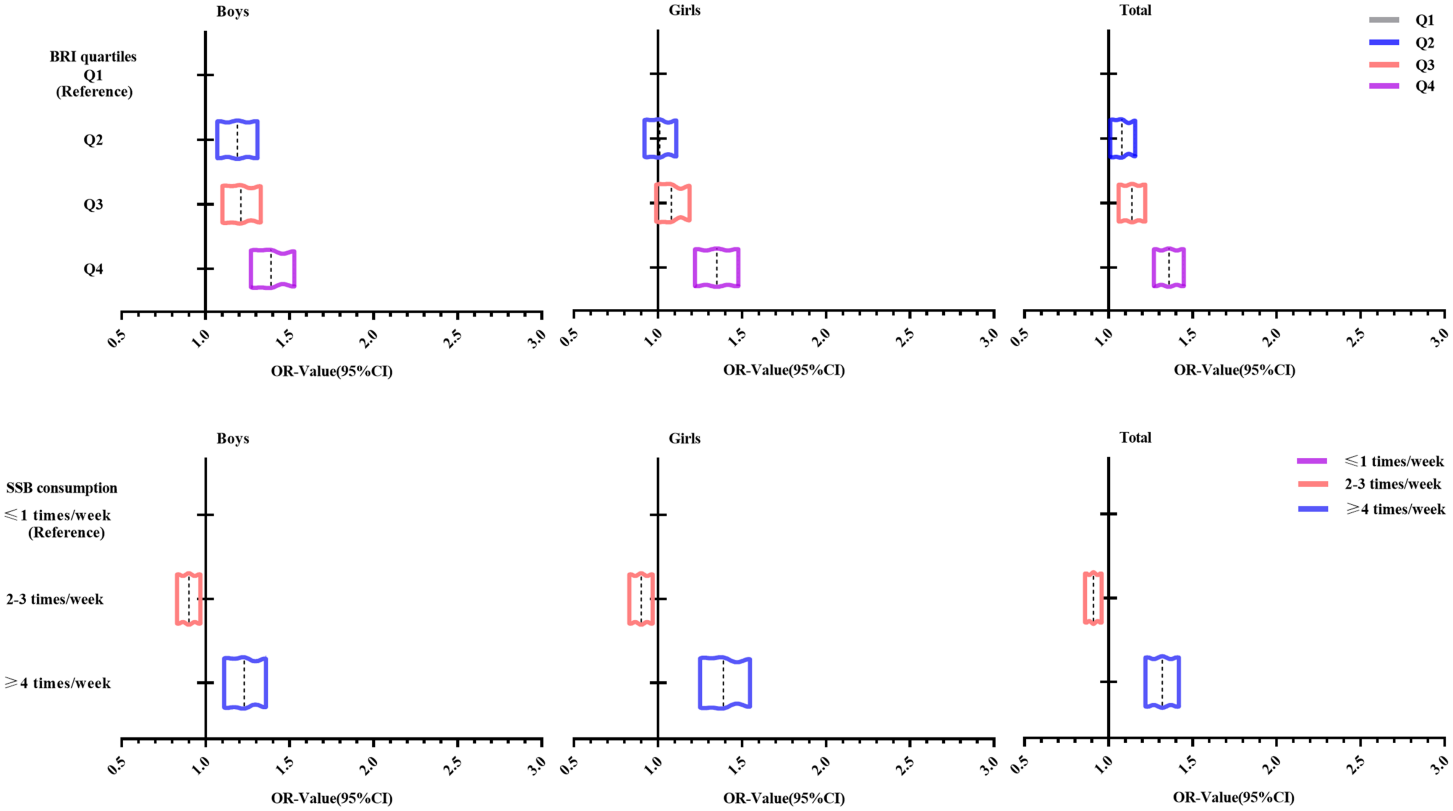
Trends in ORs of logistic regression analysis of psychological symptoms in adolescents with different BRI and SSB consumption.

Figure 2 shows the trend of OR values for logistic regression analysis of the prevalence of psychological symptoms in adolescents with different BRI and SSB consumption. Overall, it can be seen that the OR values of adolescents in the BRI quartiles for the Q4 group and BRI quartiles ≥4 times/week group were larger and shifted more to the right.

Table 4 shows the ordered logistic regression analysis of different BRI and SSB consumption and psychological symptoms in adolescents. Ordered logistic regression analyses were conducted with the presence of psychological symptoms as the dependent variable and BRI and SSB consumption as the independent variables in Chinese adolescents. The analytical model was adjusted age, father’s education, mother’s education, mode of commuting to and from school, sleep duration, and MVPA. The results show that after adjusting for relevant covariates, ordered logistic regression analysis showed that with BRI quartiles Q1 and SSB consumption ≤1 times/week as the reference group, the adolescents in the group with BRI quartiles of Q4 and SSB consumption ≥4 times/week (OR = 2.01, 95% CI: 1.77–2.30) had the highest risk of developing psychological symptoms (*p* < 0.001). The same trend was observed in boys (OR = 1.77, 95% CI: 1.48 to 2.13) and girls (OR = 2.61, 95% CI: 2.13 to 3.20) (*p* < 0.001).

**Table 4:** Ordered logistic regression analysis of psychological symptoms in Chinese adolescents with different BRI and SSB consumption.

Sex	Classification of interaction		Psychological symptoms	
	BRI quartiles	SSB consumption	OR (95% CI)	*p*-value
Boys	Q1	≤1 times/week	1.00	
		2–3 times/week	0.85 (0.72 ~ 1.00)	0.054
		≥4 times/week	1.37 (1.12 ~ 1.67)	0.002
	Q2	≤1 times/week	1.11 (0.93 ~ 1.33)	0.260
		2–3 times/week	1.06 (0.90 ~ 1.24)	0.506
		≥4 times/week	1.52 (1.24 ~ 1.85)	<0.001
	Q3	≤1 times/week	1.21 (1.02 ~ 1.45)	0.032
		2–3 times/week	1.04 (0.88 ~ 1.22)	0.676
		≥4 times/week	1.43 (1.17 ~ 1.74)	<0.001
	Q4	≤1 times/week	1.27 (1.07 ~ 1.51)	0.007
		2–3 times/week	1.21 (1.04 ~ 1.42)	0.015
		≥4 times/week	1.77 (1.48 ~ 2.13)	<0.001
Girls	Q1	≤1 times/week	1.00	
		2–3 times/week	0.88 (0.76 ~ 1.01)	0.068
		≥4 times/week	1.41 (1.16 ~ 1.71)	0.001
	Q2	≤1 times/week	0.92 (0.79 ~ 1.07)	0.283
		2–3 times/week	0.95 (0.83 ~ 1.10)	0.489
		≥4 times/week	1.30 (1.06 ~ 1.59)	0.012
	Q3	≤1 times/week	1.05 (0.90 ~ 1.22)	0.527
		2–3 times/week	0.97 (0.84 ~ 1.12)	0.701
		≥4 times/week	1.54 (1.25 ~ 1.88)	<0.001
	Q4	≤1 times/week	1.25 (1.07 ~ 1.45)	0.005
		2–3 times/week	1.19 (1.03 ~ 1.38)	0.016
		≥4 times/week	2.61 (2.13 ~ 3.20)	<0.001
Total	Q1	≤1 times/week	1.00	
		2–3 times/week	0.86 (0.77 ~ 0.96)	0.008
		≥4 times/week	1.39 (1.21 ~ 1.59)	<0.001
	Q2	≤1 times/week	0.99 (0.88 ~ 1.12)	0.924
		2–3 times/week	1.00 (0.90 ~ 1.11)	0.969
		≥4 times/week	1.42 (1.23 ~ 1.63)	<0.001
	Q3	≤1 times/week	1.12 (1.00 ~ 1.25)	0.055
		2–3 times/week	1.00 (0.90 ~ 1.12)	0.936
		≥4 times/week	1.47 (1.28 ~ 1.69)	<0.001
	Q4	≤1 times/week	1.26 (1.12 ~ 1.41)	<0.001
		2–3 times/week	1.21 (1.09 ~ 1.34)	<0.001
		≥4 times/week	2.01 (1.77 ~ 2.30)	<0.001

OR, Odds Ratio; 95% CI, 95% Confidence Interval. BRI, body roundness index; SSB, sugar-sweetened beverage. The analytical model was adjusted age, father’s education, mother’s education, mode of commuting to and from school, sleep duration, and MVPA.

**Figure 3:**
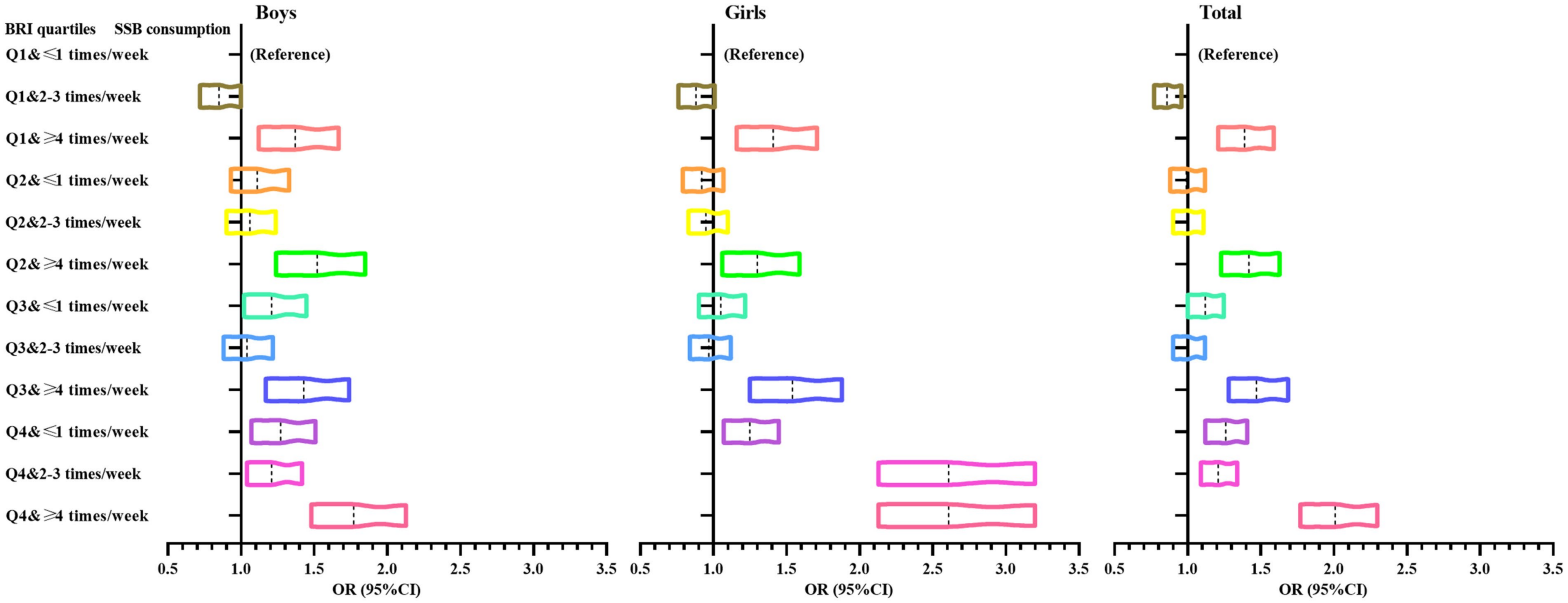
Trends in ORs of ordered logistic regression analysis of psychological symptoms in adolescents with different BRI and SSB consumption.

Figure 3 shows the trend of OR values for ordered logistic regression analysis of the prevalence of psychological symptoms in adolescents with different BRI and SSB consumption. Overall, it can be seen that adolescents in the group with BRI quartiles of Q4 and in the group with BRI quartiles ≥4 times/week had the largest ORs, and shifted more to the right. The same trend was observed for both boys and girls.

## 4 Discussion

The occurrence of psychological symptoms poses a serious threat to the health of adolescents and has become an important mental health issue of concern to countries around the world (40). The present study showed that the prevalence of psychological symptoms among Chinese adolescents was 19.2%, which was higher than the results of the survey (15%) (41), but also lower than the findings on adolescents in developing countries (32.4%) (42). China is a developing country, and with economic development, the lifestyles of Chinese adolescents have changed considerably, focusing on the decline in the level of physical activity, the prolongation of screen time, and static behaviors, which has led to a rapid rise in obesity rates and a series of negative impacts on adolescent physical and mental health (43). Coupled with the fact that the middle school stage is at the peak of adolescent development, the mental health of adolescents at this stage is extremely unstable, which leads to a higher prevalence of psychological symptoms. Surveys show that the prevalence of depressive symptoms among Chinese adolescents is 19.85%, with large differences between the central and western parts of the country, ranging from 14.75–24.96% overall (44). In addition, this, coupled with the effects of academic stress at the secondary school level, further increases the risk of adolescents with psychological symptoms (45). However, as people continue to pay more attention to mental health, the prevalence of psychological symptoms among Chinese adolescents is lower than that of other developing countries. According to a survey, the prevalence of psychological symptoms among adolescents in the African country of Tanzania is as high as 51.9%, which is significantly higher than that of Chinese adolescents (46). In conclusion, the prevalence of psychological symptoms among Chinese adolescents is still high, and relevant research should be conducted on the factors affecting them to minimize the continued rise of psychological symptoms.

The results of this study also showed that the BRI of Chinese adolescents was (2.03 ± 0.94), a result that was low compared to the results of related studies (3.229, 47). The reasons for this are associated with the different groups investigated in different studies and the different ages of the participants. The results of the present study also showed that the number of adolescents with SSB consumption ≥4 times/week was 14.7%, indicating that the level of SSB consumption among Chinese adolescents is relatively high, which should be given attention and concern. In terms of the association with psychological symptoms, the results of this study showed that the higher the BRI value, the higher the positive association with the occurrence of psychological symptoms among adolescents in the Q4 group. It indicates that as the BRI value increases, the prevalence of psychological symptoms among adolescents shows an increasing trend. The results of this study also showed that the prevalence of psychological symptoms was higher in adolescents in the higher SSB consumption group, with a positive correlation between the two. Past studies have confirmed that those with higher BRI have a higher risk of depression and anxiety (47). However, the results are not entirely consistent. Other studies have confirmed that there is no significant association between BRI and depression and anxiety (48). It has also been demonstrated that higher SSB consumption is positively associated with the development of obesity, which leads to an increased risk of psychological symptoms that can lead to the development of mental health problems (49). A study of adolescents showed that higher SSB consumption tended to lead to changes in the microbiology of the gut flora, which can cause hormonal disturbances in the brain, leading to emotional problems and negative effects on mental health (50). It has also been found that obese people are relatively less physically active, and that obesity-induced declines in self-confidence, which can lead to social disorders, can also hurt mental health development (51). Both the increase in BRI and the increase in SSB consumption in the present study led to a higher risk of obesity development, which in turn caused an increase in the prevalence of psychological symptoms.

It is noteworthy that Chinese adolescents with SSB consumption 2–3 times/week were negatively associated with the occurrence of psychological symptoms in the present study, a result that differs from that of previous studies (52, 53). Past studies have confirmed that SSB consumption in adolescents can prompt the brain to secrete dopamine, which brings about a transient feeling of euphoria, prompting a happy mood, and can alleviate negative psychological effects to a certain extent, thus playing a positive role in the development of mental health (54, 55). It can be inferred that appropriate SSB consumption may alleviate the occurrence of psychological symptoms in adolescents and have a positive impact on their mental health. The results of the ordered logistic regression analyses in this study showed that adolescents with higher BRI and higher SSB consumption were significantly and positively associated with the occurrence of psychological symptoms and the same trend was found in both boys and girls. Thus, in the process of psychosocial symptoms intervention in Chinese adolescents, special attention should be paid to the effective control of BRI, to keep it within a reasonable range. At the same time, the excessive consumption of SSB should be prevented.

There are certain strengths and limitations of this study. Strengths: First, to the best of our knowledge, this study analyzed for the first time the existence of associations between BRI and SSB consumption and psychological symptoms in Chinese adolescents. This study provides necessary references and lessons for Chinese adolescents’ psychological symptoms interventions. Second, as China is a vast country with large regional differences, this study selected adolescent participants from six geographical regions in China, which is a relatively large sample size and representative of the population. However, this study also has certain limitations. First, the present study was a cross-sectional survey, which was only able to understand the associations between BRI and SSB consumption and psychological symptoms among Chinese adolescents, but not the causal associations between them. Prospective cohort studies should be conducted in the future to better analyze the causal associations. Second, the limited number of covariates included in the analysis of this study may have affected the results. In the future, more covariates should be included, such as smoking, drinking, and exercise habits, to more accurately analyze the association between BRI and SSB consumption and psychological symptoms. Third, although the present study investigated participants from six geographic regions in China, because significant regional differences in lifestyle, dietary behaviors, or psychological symptoms may limit the applicability of this study’s findings to other populations, especially in other countries or non-urban environments, and more extensive research should be conducted in the future. Fourth, the survey of SSB consumption in this study was obtained by participants’ self-assessment using a questionnaire, which may have some potential bias from the actual SSB consumption. In the future, more objective assessment methods should be incorporated for assessment to improve the reliability of the study.

## 5 Conclusion

The results of this study found that there is an association between BRI and SSB consumption and psychological symptoms in Chinese adolescents. Both increased BRI and increased SSB consumption led to an increased prevalence of psychological symptoms in adolescents. In addition, it is worth noting that moderate SSB consumption may reduce the prevalence of psychological symptoms, which warrants more extensive investigation and research in the future. Based on the results of this study, we suggest that adolescent SSB consumption should be moderately controlled, while BRI should be effectively reduced to keep it in a reasonable range. The results of this study provide a necessary reference for the prevention and intervention of psychological symptoms in Chinese adolescents.

## Data Availability

The raw data supporting the conclusions of this article will be made available by the authors, without undue reservation.
